# A sport inspired kabaddi game optimizer for accurate parameter estimation of solar photovoltaic models

**DOI:** 10.1038/s41598-025-32437-5

**Published:** 2026-01-21

**Authors:** Tummala S. L. V. Ayyarao, G. Indira Kishore, Ark Dev, U. Siddaraj

**Affiliations:** 1 Department of Electrical and Electronics Engineering, GMR Institute of Technology, Rajam, 532127 Andhra Pradesh India; 2 Department of Electrical Engineering, Parul Institute of Engineering and Technology, Parul University, Vadodara, 391760 Gujarat India; 3 Department of Electrical and Electronics Engineering, Manipal Institute of Technology, Manipal Academy of Higher Education, Manipal, 576104 Karnataka India

**Keywords:** Solar PV system, Diode models, Kabaddi game optimizer, Parameter estimation, Optimization algorithm, Energy science and technology, Engineering, Mathematics and computing

## Abstract

This paper proposes a Kabaddi Game Optimizer (KGO), a sport-inspired metaheuristic for accurate solar photovoltaic (PV) modelling. KGO models Kabaddi strategies (Dubki and Akraman), adaptive weights and weak-player replacement to balance exploration and exploitation. Its performance is first validated on the CEC 2017 benchmark set against seven well-known optimizers, where KGO consistently attains the best average rank. KGO is then coupled with the Newton–Raphson method to estimate parameters of single, double, and triple diode PV models and a PWP-201 PV module. Using RTC France cell and module data, KGO achieves RMSE values of 7.729857E−04, 7.43146E−04, 7.3771E−04, and 2.0529E−03 for the single-, double-, and triple-diode models, and the PV module, respectively, demonstrating accurate, robust, and fast PV parameter estimation.

## Introduction

Sustainable energy solutions are crucial today to combat climate change, ensure long-term energy security, reduce environmental degradation, and support global economic and social well-being. Solar energy is crucial as a sustainable solution because it provides a clean, renewable, and abundant power source that mitigates climate change and reduces our dependence on finite fossil fuels. Thus, solar energy installations are rapidly increasing worldwide and are expected to continue growing, as declining costs and supportive policies drive solar power toward becoming a dominant source in global electricity markets.

Solar PV modelling is vital for accurately predicting energy output, optimizing system design, evaluating performance under varying conditions, and guiding cost-effective integration of solar power into the energy grid. Several types of solar PV models commonly studied in academic literature include the SDM, DDM, TDM, their modified versions, and data-driven machine learning models, each designed to predict solar panel performance under different conditions in a simple and practical manner. Finding the optimal parameters for a solar PV model is essential for accurately representing a physical PV system, which in turn leads to a precise prediction of its performance, thereby enabling effective system design, analysis, and control. In this regard, optimization plays a crucial role.

Optimization algorithms can be broadly categorized into two categories: deterministic and metaheuristic. Deterministic optimization methods apply a fixed, repeatable set of rules, ensuring that identical starting conditions always produce the same outcome. They are often gradient-based, meaning they use the derivative of the objective function to find a solution. However, such methods are susceptible to convergence at local optima—solutions that may be satisfactory yet fall short of representing the global optimum. On the other side, metaheuristic algorithms are inspired by natural or physical processes and use random factors to explore the solution space. They are frequently effective at bypassing local optima and achieving satisfactory solutions within reasonable timeframes, even when addressing complex problems. Metaheuristic algorithms can be broadly classified into categories such as human-based, physics-based, swarm-based, and evolutionary-based approaches, depending on the principles or phenomena they are inspired by. Evolutionary metaheuristic algorithms are optimization techniques inspired by natural evolution. Swarm-based algorithms are optimization techniques inspired by the collective behaviour of social animals to identify optimal solutions. Physics-based algorithms are those inspired by or modelled on physical processes. Human-based algorithms are inspired by human behaviour, decision-making, and problem-solving strategies. Table 1 lists the algorithms applied for solar PV modelling.

**Table 1:** Summary of algorithms applied in solar PV modelling.

References	Algorithm	Category	Inspiration	Novelty/remarks
^1^	Differential Evolution	Evolutionary	Natural evolution	The method can compute model parameters at any irradiance and temperature, using manufacturer datasheet information
^2^	Genetic Algorithm	Evolutionary	Biological evolution	The paper uses a genetic algorithm to find the best parameters for two different solar cell models
^3^	PSO	Swarm-based	Social behavior of animals	The paper offers a comprehensive review of PSO, highlighting its ability to operate directly in continuous real number space
^4^	Flower pollination algorithm		Pollination process of flowering plants	Accurately extracts parameters for single- and double-diode PV models, works well with different data sources
^5^	Bacterial Foraging		The foraging behavior of bacteria	The paper proposes using BFA to accurately model solar PV characteristics
^6^	Simulated Annealing	Physics-based	Metallurgical process of annealing	The paper proposes a Simulated Annealing approach to extract parameters for single-diode, two-diode, and PV module models
^7^	Wind-Driven optimization		The movement of air particles in the atmosphere	The paper proposes Wind-Driven Optimization for double-diode solar cell parameter extraction, showing superior performance across multiple datasets
^8^	Kepler optimization		Planetary motion and celestial mechanics	The basic algorithm is upgraded with a local escaping operator to avoid local optima
^9^	War strategy optimization	Human based	Ancient war strategies	The optimization algorithm is combined with NR method for optimal parameter estimation
^10^	Teaching learning optimization		Learning process	The basic algorithm is enhanced with dynamic oppositional learning and sorting-based mechanism

Recent works:

To address the drawbacks of the basic genetic algorithm and PSO, an enhanced hybrid algorithm combining the genetic algorithm and PSO is proposed in^11^ for accurate parameter estimation. An improved neural network–based optimization algorithm employing reinforcement learning and adaptive strategies is used for more accurate and reliable parameter extraction of PV models^12^. Ridha proposed a modified version of arithmetic optimization algorithm by integrating the basic algorithm with NR method and Levenberg–Marquardt operator^26^. A Puma-based optimization approach is proposed to accurately extract the nine unknown parameters of the TDM, demonstrating superior accuracy and robustness compared with several existing algorithms across different PV modules and operating conditions^13^.

The existing algorithms employed for solar PV modelling are associated with two major concerns. The first involves the limitations of the algorithms adopted in existing studies, while the second arises from the nonlinear nature of the PV equations and the resulting challenges in defining an appropriate objective function.

When modelling solar PV systems using metaheuristic algorithms, several challenges may arise, including the tendency of some algorithms to become trapped in local optima, the need for careful tuning of algorithm-specific parameters, and high computational demands. In addition, slow convergence in certain algorithms limits their effectiveness in handling complex PV models. To address these challenges, several research directions have been explored in the literature, including:

Develop new algorithms with faster convergence and higher efficiency, in line with the no free-lunch theorem^14^, which implies continuous scope for new algorithms.

Enhance existing algorithms by incorporating features such as learning strategies^15–17^, improved operators^18–20^, and adaptive mechanisms^21,22^. Some of the examples of learning strategies include generalized oppositional based TLBO^15^, orthogonal based learning^16^.

The hybridization of algorithms by combining complementary features of multiple optimization techniques to improve overall performance and robustness.

The PV current equation is highly nonlinear in nature. However, some researchers simplify this nonlinearity to obtain an objective function. As a result, the value of the error function does not necessarily correspond to the actual current error, preventing the error function from accurately representing the current mismatch. Despite this limitation, most meta-heuristic algorithms reported in the literature rely on such error formulations, and their results may therefore not reliably achieve the intended accuracy. Most existing algorithms are limited to the SDM and DDM, and very limited research has been conducted on the TDM and PV module with comprehensive convergence analysis.

To overcome the issues of metaheuristic algorithms in solar PV modelling, this paper proposes a novel sport-inspired Kabaddi Game Optimizer (KGO). The proposed algorithm is inspired by the sport of Kabaddi, which is popular in South Asia, and is mathematically modelled based on it. To enhance the exploration and exploitation capabilities, two gaming strategies (Dubki and Akraman) are incorporated into the algorithm. Additionally, a nonlinear adaptive weight updating scheme is introduced to achieve faster convergence. To address the nonlinearity in the PV current equation, the proposed KGO algorithm is integrated with the NR method to resolve the nonlinearities in the PV model.

The core innovations of this research are presented as follows:

5. A novel metaheuristic optimization algorithm, inspired by the Kabaddi game and formulated with two strategic components, is proposed in this paper.
6. The proposed KGO algorithm is first applied to the CEC 2017 benchmark test suite and compared with popular algorithms from the literature.
7. The algorithm is combined with the Newton–Raphson approach to achieve optimal parameter estimation for SDM, DDM, and TDM models.

The remaining paper is outlined as follows: In “Mathematical model of solar PV system”, different diode models of solar PV system is mathematically described along with the objective function. Section “Kabaddi game optimizer” describes the novel KGO algorithm with flowchart. Section “Results and discussion” deals with validation of KGO algorithm on the CEC 2017 benchmark suite and its application for solar PV system parameter estimation. Finally, the article ends with few conclusive remarks and scope for future work.

## Mathematical model of solar PV system

### Single diode model

In photovoltaic studies, the single-diode model is valuable because it reduces complexity while still reflecting a solar cell’s main electrical features^23^. With this model, the prediction for the performance of solar cell under various operating conditions can be obtained. It can be applied in design and progress of solar cell systems along with its parameter evaluation^24–26^.

**Fig. 1:**
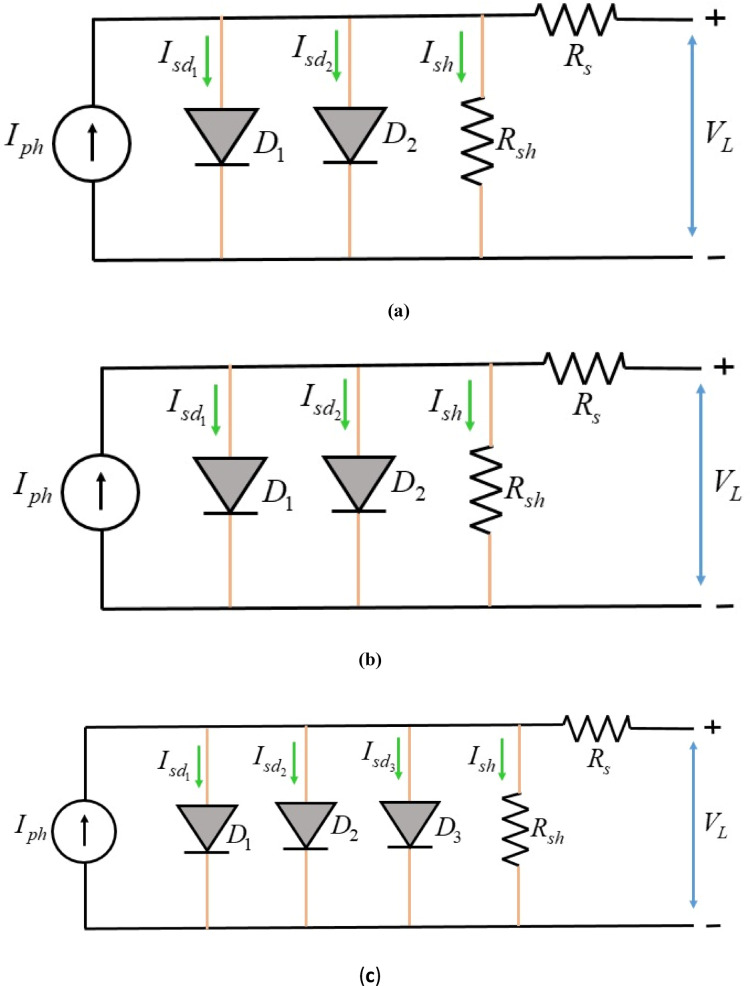
Equivalent circuit representations of photovoltaic cell models: (**a**) SDM (**b**) DDM, and (**c**) TDM.

By applying Kirchhoff current law is applied to circuit in Fig. 1a. The expression for load current is represented as:

1 $$\:{I}_{L}={I}_{ph}-{I}_{d}-{I}_{sh}$$

The equations of $\:{I}_{d}\:\&\:\:{I}_{sh}$:

2 $$\:{I}_{d}={I}_{sd}\left({e}^{\frac{q\left\{{V}_{L}+{I}_{L}{R}_{S}\right\}}{nkT}}-1\right)$$

3 $$\:{I}_{sh}=\frac{{V}_{L}+{I}_{L}{R}_{S}}{{R}_{sh}}$$

The equations for $\:{I}_{L}\:$is obtained by replacing the terms for $\:{I}_{d}\:and\:{I}_{sh}$ from (2) and (3) in (1), and is expressed as:

4 $$\:{I}_{L}={I}_{ph}-{I}_{sd}\left({e}^{\frac{q\left\{{V}_{L}+{I}_{L}{R}_{S}\right\}}{nkT}}-1\right)-\frac{{V}_{L}+{I}_{L}{R}_{S}}{{R}_{sh}}$$

Apart from the established parameters, five unknown quantities {$\:{I}_{ph},\:{I}_{sd},{R}_{sh},\:{R}_{s},\:n\}\:\:$ must be estimated^17^.

### Double diode model

From Fig. 1b, the load current can be obtained from the equivalent circuit of DDM and expressed as:

5 $$\:{I}_{L}={I}_{ph}-{I}_{d1}-{I}_{d2}-{I}_{sh}$$

Following an approach analogous to the SDM, the output current of a PV cell can be expressed as:

6 $$\:{I}_{L}={I}_{ph}-{I}_{sd1}\left({e}^{\frac{q\left\{{V}_{L}+{I}_{L}{R}_{S}\right\}}{{n}_{1}kT}}-1\right)-{I}_{sd2}\left({e}^{\frac{q\left\{{V}_{L}+{I}_{L}{R}_{S}\right\}}{{n}_{2}kT}}-1\right)-\frac{{V}_{L}+{I}_{L}{R}_{S}}{{R}_{sh}}$$

To complete the model, seven unknown parameters {$\:{I}_{ph},\:{I}_{sd1},{I}_{sd2},{R}_{sh},\:{R}_{s},{n}_{1},{n}_{2}\}$ need to be determined along with the known quantities.

### Triple diode model

From Fig. 1c, $\:{I}_{L}$of TDM can be derived and mentioned as:

7 $$\:{I}_{L}={I}_{ph}-{I}_{d1}-{I}_{d2}{-I}_{d3}-{I}_{sh}$$

Extending the methodology of the SDM, the final expression for the output current of the PV cell is obtained as:

8 $$\:{I}_{L}={I}_{ph}-{I}_{sd1}\left({e}^{\frac{q\left\{{V}_{L}+{I}_{L}{R}_{S}\right\}}{{n}_{1}kT}}-1\right)-{I}_{sd2}\left({e}^{\frac{q\left\{{V}_{L}+{I}_{L}{R}_{S}\right\}}{{n}_{2}kT}}-1\right)-{I}_{sd3}\left({e}^{\frac{q\left\{{V}_{L}+{I}_{L}{R}_{S}\right\}}{{n}_{3}kT}}-1\right)-\frac{{V}_{L}+{I}_{L}{R}_{S}}{{R}_{sh}}$$

In the TDM, a total of nine parameters remain unknown and require estimation, namely: {$\:{I}_{ph},\:{I}_{sd1},{I}_{sd2},\:{I}_{sd3},\:{R}_{sh},\:{R}_{s},{n}_{1},{n}_{2},\:{n}_{3}\}$.

### PV module

The output current of a solar PV module is expressed mathematically as:

9 $$\:{I}_{L}={I}_{ph}{N}_{p}-{I}_{sd}{N}_{p}\left({e}^{\left\{\frac{q\left\{{V}_{L}+\frac{{I}_{L}{R}_{S}{N}_{S}}{{N}_{p}}\right\}}{n{N}_{S}kT}\right\}}-1\right)-\frac{{V}_{L}+\frac{{I}_{L}{R}_{S{N}_{S}}}{{N}_{p}}}{\frac{{R}_{sh}{N}_{s}}{{N}_{p}}}$$

In series-connected configurations of solar cells, the number of parallel cells is considered unity $\:({N}_{p}=1)$. Consequently, the expression for the output load current of the PV module reduces to:

10 $$\:{I}_{L}={I}_{ph}-{I}_{sd}\left({e}^{\left\{\frac{q\left\{{V}_{L}+{I}_{L}{R}_{S}{N}_{S}\right\}}{n{N}_{S}kT}\right\}}-1\right)-\frac{{V}_{L}+{I}_{L}{R}_{S}{N}_{S}}{{R}_{sh}{N}_{s}}$$

Based on the equivalent circuit representation, the load current of the three models can be calculated using Eqs. (11)–(13).

11 $$\:{I}_{ph}-{I}_{sd}\left({e}^{\frac{q\left\{{V}_{L}+{I}_{L}{R}_{S}\right\}}{nkT}}-1\right)-\frac{{V}_{L}+{I}_{L}{R}_{S}}{{R}_{sh}}-{I}_{L}=0$$

12 $$\:{I}_{ph}-{I}_{sd1}\left({e}^{\frac{q\left\{{V}_{L}+{I}_{L}{R}_{S}\right\}}{{n}_{1}kT}}-1\right)-{I}_{sd2}\left({e}^{\frac{q\left\{{V}_{L}+{I}_{L}{R}_{S}\right\}}{{n}_{2}kT}}-1\right)-\frac{{V}_{L}+{I}_{L}{R}_{S}}{{R}_{sh}}-{I}_{L}=0$$

13 $$\:{I}_{ph}-{I}_{sd1}\left({e}^{\frac{q\left\{{V}_{L}+{I}_{L}{R}_{S}\right\}}{{n}_{1}kT}}-1\right)-{I}_{sd2}\left({e}^{\frac{q\left\{{V}_{L}+{I}_{L}{R}_{S}\right\}}{{n}_{2}kT}}-1\right)-{I}_{sd3}\left({e}^{\frac{q\left\{{V}_{L}+{I}_{L}{R}_{S}\right\}}{{n}_{3}kT}}-1\right)-\frac{{V}_{L}+{I}_{L}{R}_{S}}{{R}_{sh}}-{I}_{L}=0$$

With measurements of current and voltage, the estimated current is attained as:

14 $$\:{I}_{L.calc}={I}_{ph}-{I}_{sd}\left({e}^{\left(\frac{q\left\{{V}_{L.mes}+{I}_{L,mes}{R}_{S}\right\}}{nkT}\right)}-1\right)-\frac{{V}_{L.mes}+{I}_{L.mes}{R}_{S}}{{R}_{sh}}$$

Substituting Eq. (13) into Eq. (9), will result in:

15 $$\:{F}_{obj}=\sqrt{\frac{1}{N}\left(\sum\:_{i=1}^{N}{\left[{I}_{L.mes}-\left\{{I}_{ph}-{I}_{sd}\left({e}^{\left(\frac{q\left\{{V}_{L.mes}+{I}_{L,mes}{R}_{S}\right\}}{nkT}\right)}-1\right)-\frac{{V}_{L.mes}+{I}_{L.mes}{R}_{S}}{{R}_{sh}}\right\}\right]}^{2}\right)}$$

whereas for DDM,

16 $$\:{F}_{obj}=\sqrt{\frac{1}{N}\left(\sum\:_{i=1}^{N}{\left[{I}_{L.mes}-\left\{\begin{array}{c}{I}_{ph}-{I}_{sd1}\left({e}^{\frac{q\left\{{V}_{L,mes}+{I}_{L},mes{R}_{S}\right\}}{{n}_{1}kT}}-1\right)\\\:-{I}_{sd2}\left({e}^{\frac{q\left\{{V}_{L,mes}+{I}_{L,mes}{R}_{S}\right\}}{{n}_{2}kT}}-1\right)-\frac{{V}_{L.mes}+{I}_{L.mes}{R}_{S}}{{R}_{sh}}\end{array}\right\}\right]}^{2}\right)}$$

The high degree of nonlinearity in Eq. (11) implies that replacing $\:{{I}_{L}\:with\:I}_{L,mes}$​ directly may compromise accuracy, leading the objective function to converge to an erroneous model.

Therefore, this nonlinear equation must be solved to identify the parameters of the solar PV model^27^. The Newton-Raphson Method, an iterative approach that is very popular, can be applied for this purpose. Using the derivative of the function, it refines the approximation successively to achieve rapid convergence. Starting from an initial guess $\:{x}_{0}$, the estimate is refined by applying the gradient of the function at that point. The KGO algorithm works in coordination with the NR method for solving the nonlinear equations.

The following nonlinear equation will compute the current for basic SDM:

17 $$\:f\left(x\right)={I}_{ph}-{{I}_{sd}e}^{\left(\frac{q\left\{{V}_{L}+x{R}_{S}\right\}}{nkT}-1\right)}-\frac{{V}_{L}+x{R}_{S}}{{R}_{sh}}-x$$

In the case of the DDM, the corresponding nonlinear equation is formulated as follows:

18 $$\:{g\left(x\right)=I}_{ph}-{I}_{sd1}\left({e}^{\frac{q\left\{{V}_{L}+x{R}_{S}\right\}}{{n}_{1}kT}}-1\right)-{I}_{sd2}\left({e}^{\frac{q\left\{{V}_{L}+x{R}_{S}\right\}}{{n}_{2}kT}}-1\right)-\frac{{V}_{L}+x{R}_{S}}{{R}_{sh}}-x$$

The variable *x* is determined using the nonlinear relation defined in Eq. (17). The derivative required for $\:{f}^{{\prime\:}}\left(x\right)$ the iterative solution is expressed as:

19 $$\:{f}^{{\prime\:}}\left(x\right)=-{{I}_{sd}\frac{q{R}_{S}}{nkT}e}^{\left(\frac{q\left\{{V}_{L}+x{R}_{S}\right\}}{nkT}\right)}-\frac{{R}_{S}}{{R}_{sh}}-1$$

Iterations are carried out until either the change between consecutive estimates is sufficiently small or the process reaches the specified iteration limit. In the NR method, the function is locally linearized using its tangent at the present estimate, and the root of this linear form is then computed to update the solution.

Following k iterations, the solution can be expressed as:

20 $$\:{x}_{k+1}={x}_{k}-\frac{f\left({x}_{k}\right)}{{f}^{{\prime\:}}\left({x}_{k}\right)}$$

The iteration stops once $\:\left|f\left(x\right)\right|<\epsilon\:$ is satisfied or when the predefined maximum iteration limit is reached. In the optimization process, the Newton–Raphson method computes the objective function by solving the nonlinear Eq. (18) for a given voltage, using the PV parameters supplied by the optimization algorithm, to obtain the corresponding current.

**Fig. 2:**
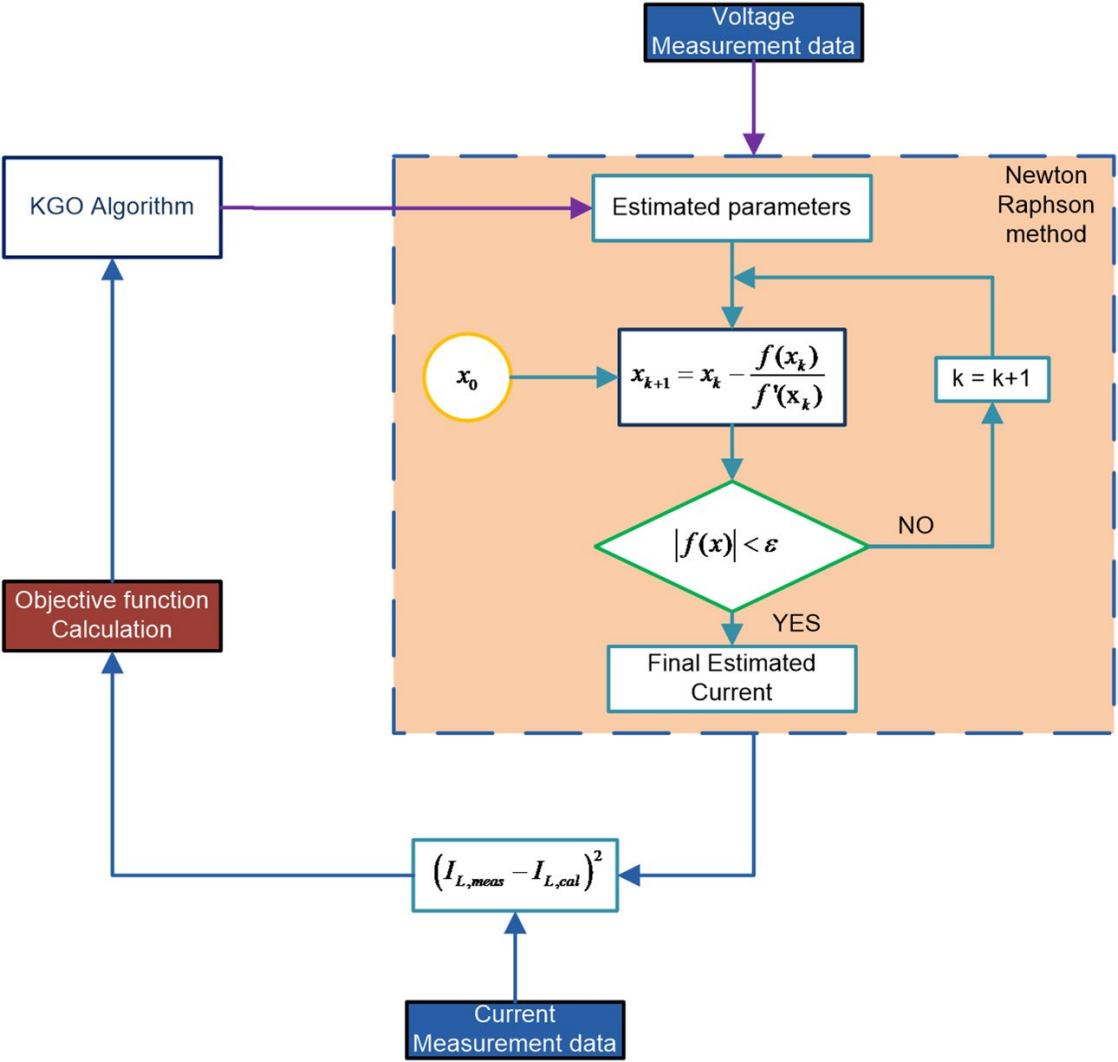
Framework for solar PV parameter estimation using the NR method combined with Kabaddi Game Optimizer.

Figure 2 summarises the proposed framework. Measured I–V data and the mathematical PV models (“Mathematical model of solar PV system”) are used to define the RMSE-based objective function. For a given parameter set, the Newton–Raphson method solves the nonlinear current equation to obtain model currents. The optimization algorithm iteratively updates the PV parameters to minimise this RMSE. The process is repeated for SDM, DDM, TDM and the PWP-201 module.

### Objective function

The primary objective is to identify the unknown parameters of PV models based on experimental measurement data. For this purpose, an objective function is constructed to minimize the RMSE between the experimental measurements and the simulated outputs. By reducing this error, the accuracy of the model is improved so that it reliably reflects the real system. The RMSE expression between measured and computed current values is formulated as:

21 $$\:{F}_{obj}=\sqrt{\frac{1}{N}\left(\sum\:_{i=1}^{N}{\left[{I}_{L.mes}-{I}_{L.calc}\right]}^{2}\right)}$$

## Kabaddi game optimizer

### Inspiration

Kabaddi is a team sport that combines both physical strength and strategic thinking. It is a game that also involves strategic and tactical planning. Kabaddi originally started out as a recreational activity in rural areas, but has since become a very competitive, professional sport. Each country plays Kabaddi in varying formats, and each has an associated National Governing Body (NGB) that manages the sport in that country. Championships are hosted by the NGBs to identify the elite players who compete on behalf of their countries at the international level. Kabaddi is especially popular in Asian countries, with India being one of its main centres.

In the early stages of its development, there were no universal rules governing the game of Kabaddi; there were no standard rules for the number of players, the number of points needed to win, or the length of time for a match. Over time, rules were implemented to create a more structured and interesting game. In its modern form, Kabaddi features new attacking techniques and defensive strategies, which have enhanced its appeal to audiences.

There are two competing teams called raiders and defenders. The goal of the raiders is to touch as many defenders as possible in a single raid by entering the defending team’s side and then returning safely to their own side in one attempt. Raiders must do this without being caught and while holding their breath during the raid. If a raider is caught before returning to their side, the raid ends.

Raiders develop strategies to tag defenders, while defenders create game plans to trap and stop the raiders from escaping. There are two ways to score points during the game. Raiders earn points by successfully tagging defenders and returning to their side safely. Defenders earn points by capturing the raider before they escape. Players often practice advanced techniques such as dubki (ducking under tackles), the side kick, lona (eliminating all players of the opposing team), and the super catch.

### Mathematical model of KGO algorithm

In the proposed algorithm, players are divided into two groups: raiders and defenders. The optimization process starts with a randomly chosen raider entering the defenders’ area, aiming to successfully “tag” at least one defender. Defenders may attempt to trap the raider by moving closely together, which represents a local optimum. However, the algorithm is programmed to strategically avoid this trap, while the players are required to follow specific game rules that define the boundaries of the search space. These constraints include staying within marked lines, a time limit, and specific canting and tackling rules.

In the sport of Kabaddi, adopting appropriate strategies is essential for achieving victory. In this study, two prominent strategies—Dubki and Akraman—are modelled. In the Dubki strategy, the raider adjusts their momentum based on the average position of the opposing team, enabling movement toward the opponents. In contrast, the Akraman strategy causes the raider to modify their momentum with respect to the opposing team’s leader while simultaneously tracking a selected defender from the same team.

Depending on the game conditions and the current positioning of the opposing players, the raider randomly selects one of these strategies during play. For a raider to successfully tag a defender and score points, a strong coordination between mental focus and physical strength is required. The raider must effectively respond to the movements of the opposing defenders, who are typically positioned in a semicircular formation on the opposite half of the court.

**DUBKI**:

In this strategy, the raider continuously observes and interprets the movements of the defending opponents. To overcome the defence, the raider will continually change his stance to match the average location of defenders. By modifying his foot placement throughout the raid, he can anticipate defensive movements and quickly change direction, thereby increasing his chances of successful evasion. The corresponding velocity update mechanism is mathematically expressed as:

22 $$\:\varDelta\:{V}_{i}\left(t+1\right)={r}_{1}\left({R}_{i}\frac{1}{N}\sum\:_{k=1}^{\frac{N}{2}}{X}_{k}-{X}_{i}\left(t\right)\right)\:\:\:\:\:\:\:for\:the\:first\:team$$

23 $$\:\varDelta\:{V}_{i}\left(t+1\right)={r}_{1}\left({R}_{i}\frac{1}{N}\sum\:_{k=\frac{N}{2}+1}^{N}{X}_{k}-{X}_{i}\left(t\right)\right)\:\:\:\:\:\:\:for\:the\:second\:team$$

where, $\:\varDelta\:{V}_{i}$ is the change in velocity of $\:{i}^{th}$ player, $\:{R}_{i}$ is the rank of $\:{i}^{th}$player, $\:{r}_{1}$ is a random value $\:\in\:\left(\mathrm{0,1}\right),\:$ $\:{X}_{i}$ is the position of $\:{i}^{th}$ player, $\:\frac{1}{N}\sum\:_{k=1}^{\frac{N}{2}}{X}_{k}=mean\left(X\right)$is the mean position of the opposite team.

The term $\:\sum\:_{k=1}^{\frac{N}{2}}{X}_{k}$ represents the combined positions of half of the defending players, which are then averaged to obtain their mean position. Through a model’s defensive mechanism, the raider’s movement will be flexible enough in meeting the current movements of the defenders. Thus, by responding to the defenders’ collective response (e.g., the “mean” position of the defenders) the raider has a better chance of evading capture than using a previous model which considers all historical behaviour of the defender and allows for adaptation to all recent actions of the defenders.

From an algorithmic perspective, the use of the mean position of nearby defenders represents an exploitation process. By focusing on the most immediate and relevant defensive information, the raider adapts its strategy based on local intelligence rather than exploring the entire court for all possible defensive configurations. This localized exploitation enhances decision-making accuracy, restricts the search space to promising regions, accelerates convergence, and increases the probability of achieving optimal solutions within the optimization framework.

**AKRAMAN**:

In this strategy, the raider systematically explores the opponent’s court to detect and evaluate vulnerable positions. The raider dynamically adjusts its velocity by considering both the behavioural characteristics of the opposing team’s leader and the influence of a randomly selected player. This adaptive mechanism achieves a balance between directed exploration and stochastic movement, thereby increasing the likelihood of identifying and exploiting weaknesses in the opponent’s defence. The process is mathematically represented as:

24 $$\:\varDelta\:{V}_{i}\left(t+1\right)={r}_{2}\odot\:{R}_{i}\left(Lea{d}_{Op}-{X}_{i}\left(t\right)\right)+{2\times\:r}_{3}\odot\:\left({X}_{rand}-{X}_{i}\left(t\right)\right)$$

where, $\:Lea{d}_{Op}$ is the leader of the opposite team, $\:{X}_{rand}$is the position of random player of the opposite team, $\:{r}_{2}$ and $\:{r}_{3}\:$are $\:{D}^{th}$ dimension random values $\:\in\:\left(\mathrm{0,1}\right)$.

By defining $\:{r}_{2}$ and $\:{r}_{3}$ as vectors rather than scalars, the algorithm applies randomness independently across all dimensions of the search space. This design supports the exploration process by encouraging the raider to diversify his movements instead of concentrating within a limited area. The influence of the opposition leader ($\:Lea{d}_{Op}$) directs the raider toward dominant defensive positions. However, the inclusion of a randomly selected player ($\:{X}_{rand}$) introduces unpredictability and expands the search horizon. The use of vector-based randomness through $\:{r}_{2}$ and $\:{r}_{3}$ further prevents the raider from adopting repetitive or predictable behaviours. Consequently, the raider can investigate multiple regions of the court, discover new opportunities, and avoid premature convergence.

A randomized selection of attack strategies is introduced to strengthen the exploration capability of the algorithm. When the algorithm only one strategy, the risk of local optimum entrapment is extremely high. To address this limitation, the players are divided into two subgroups, referred to as Team A and Team B. Each player that is assigned to their respective team will have their leader (the team member with the greatest fitness score) selected for that week of competitive play. Accordingly, the leaders of Team A and Team B are denoted as Lead A and Lead B, respectively. In this framework, the primary objective of the raider is to attack the opposing team by adopting the most effective strategy. By allowing multiple leaders to guide player movements, the algorithm introduces greater diversity and adaptability and this reduces the risk of stagnation and minimizes the likelihood of convergence to a local optimum.

**Ranking**:

At the start of the game, each player takes relatively large steps. As the game progresses, the step size decreases exponentially when the player successfully advances toward the optimum position. Furthermore, if a player successfully tackles an opponent, their score $\:{S}_{i}$ is incremented by one.

25 $$\:{S}_{i}^{new}=\left({S}_{i}+1\right)\times\:\left({F}_{i}^{old}>{F}_{i}^{new}\right)+{S}_{i}\times\:\left({F}_{i}^{old}\le\:{F}_{i}^{new}\right)$$

The rank of the $\:{i}^{th}$ player will be updated by using the below mechanism:

26 $$\:{R}_{i}^{new}={R}_{i}\left(1-\frac{{S}_{i}^{new}}{Max\_iter}\right)$$

where $\:{R}_{i}^{new}$is the updated rank,$\:\:Max\_iter$represents the maximum iteration, and $\:{S}_{i}$ is the success score.

**Replacement of weak players**:

After each iteration, the player with the lowest fitness is replaced by a randomly generated player using the following equation:

27 $$\:{X}_{w}=r\left({X}_{R1}-{X}_{R2}\right)$$

Here $\:r$ is the random number$\:\in\:\left(\mathrm{0,1}\right)$, $\:{X}_{R1},\:\:{X}_{R2}$ are the random players.

As an extension of PSO, the algorithm involves two key steps: velocity update and position update. The position of the raiders, as defined in Eq. (6), is updated using the velocity update described in Eq. (7), along with the raider’s previous position.

28 $$\:\varDelta\:{V}_{i}\left(t+1\right)=rand\times\:{V}_{i}\left(t\right)+\varDelta\:{V}_{i}\left(t+1\right)$$

29 $$\:{X}_{i}\left(t+1\right)={X}_{i}\left(t\right)+{V}_{i}\left(t+1\right)$$

**Fig. 3:**
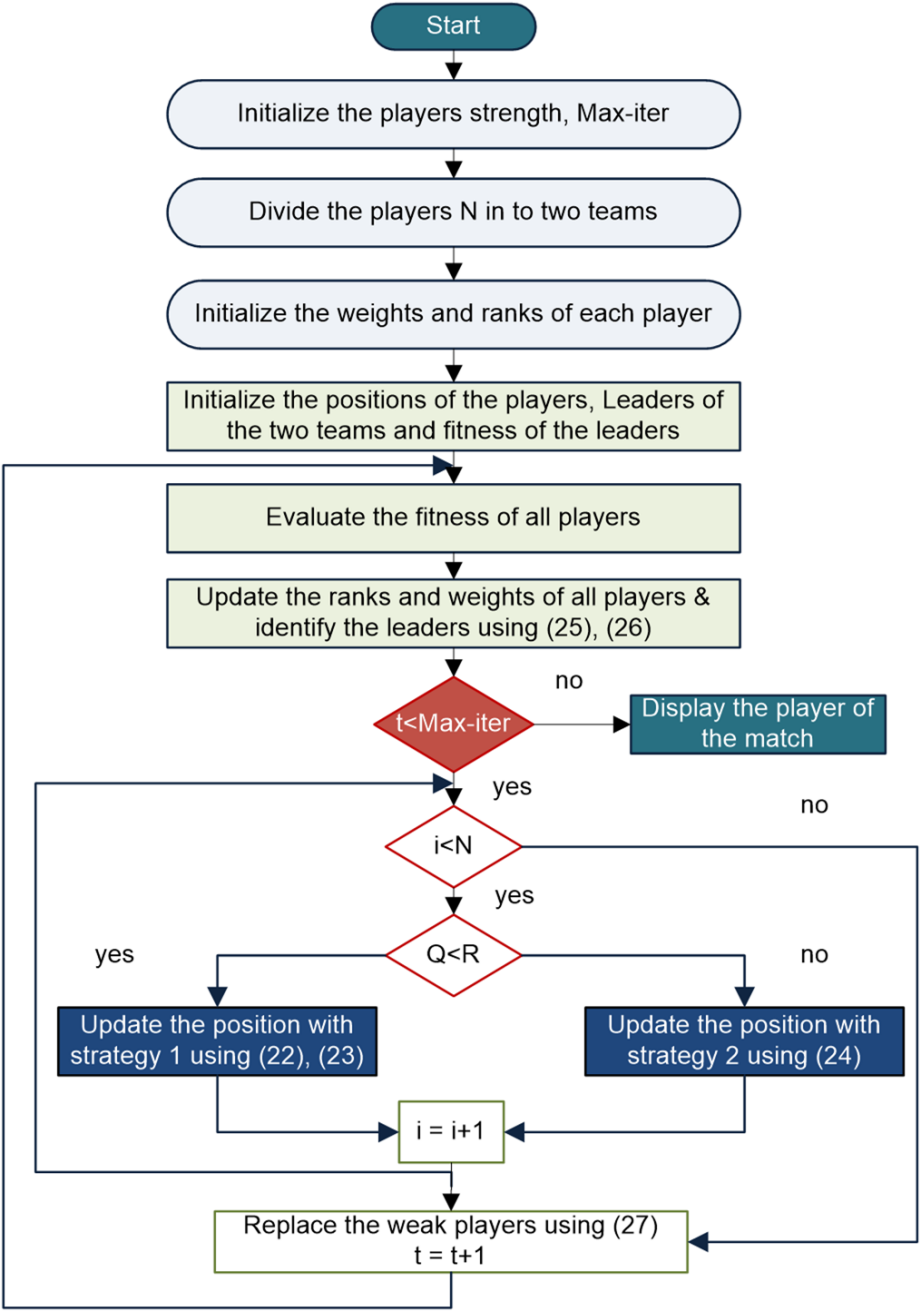
Flow diagram illustrating the KGO algorithm.

Figure 3 illustrates the step-by-step implementation of the proposed KGO algorithm.

### Application of KGO for solar PV modelling

To extract solar PV model parameters, the KGO algorithm must be used to calculate solar PV model parameters with great precision. This process has been divided into five steps:

Step 1: Start by determining how many decision variables there will be: five for SDM, seven for DDM, and nine for TDM. Next, determine the population size and maximum number of iterations.

Step 2: The next step is to create a fitness evaluation criterion. For example, the objective function is written out as the RMSE (root mean square error) between the PV device’s measured attributes and its model-predicted outputs in (15) for SDM and (16) for DDM.

Step 3: Carry out KGO algorithm operations according to the process shown in the flow diagram. At each iteration, the nonlinear PV current equation that calculates the value of the objective function must be solved with Newton-Raphson technique.

Step 4: When the maximum number of iterations has been completed, document the optimised values of the PV model parameters and the corresponding final values of the objective function.

Step 5: Using the optimal solutions, determine the I-V (current-voltage) and P-V (power-voltage) curves for each PV model and draw comparisons with physical data to evaluate the accuracy of the models.

## Results and discussion

The proposed KGO algorithm is evaluated in two cases: first, on the CEC 2017 benchmark test suite, and second, for parameter estimation of three different solar PV models.

### CEC 2017 bench

The efficacy of the proposed KGO algorithm is first verified by testing on CEC 2017 benchmark test suite with 30 different functions. The dimension of the problem is set to 30 for all the test functions. The performance of KGO is compared with PSO^28^, LSHADE, SSA^29^, WOA^30^, Chimp^31^, GWO^32^, DA^33^ and SaDE^34^. To ensure a fair comparison, all algorithms were executed with a population size of 30 and a maximum of 500 iterations and the details are given in Table 2. The statistical outcomes for all CEC 2017 benchmark functions are reported in supplementary material. A Wilcoxon rank-sum test at a 0.05 significance level was applied to compare KGO with each algorithm individually. Results confirm that KGO consistently outperformed the other algorithms. The competing algorithms were ranked based on average performance. KGO algorithm secured placed first rank, followed by SaDE in the second position. The overall rankings obtained from Friedman’s test are presented in Table 3. The complete statistical details that include the mean, median, and standard deviation for the CEC benchmark functions, are provided in the supplementary material^68^.

**Table 2:** Parameter settings of the comparative algorithms used for evaluating the performance of KGO.

Algorithm	Control Parameters	Max-Fes
SSA^29^	$\:c\in\:\left[\mathrm{2,0}\right]$	15,000
SaDE^34^	*p* = 0.05, f = 0.2	15,000
GWO^32^	$\:a\in\:\left[\mathrm{2,0}\right]$	15,000
PSO^28^	$\:{c}_{1}=2,\:{c}_{1}=2,\:\omega\:=0.3$	15,000
DA^33^	$\:c\in\:\left[\mathrm{0.1,0}\right],\:\omega\:=0.9$	15,000
L-SHADE^35^	Arc rate = 2	15,000
Chimp^31^	$\:a\in\:\left[\mathrm{2,0}\right]$	15,000
WOA^30^	$\:{a}_{1}\in\:\left[\mathrm{2,0}\right],\:b=1,{a}_{2}=\left[-2,-1\right]$	15,000

**Table 3:** Performance rankings of the proposed KGO algorithm in comparison with other competing algorithms on the CEC 2017 benchmark functions.

Function	KGO	PSO	LSHADE	SSA	WOA	Chimp	GWO	DA	SaDE
F1	4	1	6	9	7	8	3	5	2
F3	2	6	4	9	8	7	1	3	5
F4	2	4	5	9	7	8	3	6	1
F5	1	5	3	9	7	8	6	4	2
F6	3	5	4	9	7	8	2	6	1
F7	3	6	5	9	7	8	1	4	2
F8	2	6	4	9	7	8	1	5	3
F9	3	6	4	9	7	8	2	5	1
F10	1	6	4	9	8	7	5	2	3
F11	1	6	5	9	7	8	3	4	2
F12	1	4	6	9	7	8	2	5	3
F13	3	1	6	9	7	8	2	4	5
F14	1	4	6	9	8	7	3	5	2
F15	2	1	5	9	7	8	4	3	6
F16	1	5	3	9	7	8	6	4	2
F17	1	6	5	9	7	8	3	4	2
F18	1	3	6	9	8	7	2	4	5
F19	2	1	6	9	7	8	3	5	4
F20	1	6	5	9	8	7	2	4	3
F21	2	6	4	9	7	8	1	5	3
F22	1	6	2	9	7	8	3	4	5
F23	1	5	3	7	6	8	9	4	2
F24	1	5	3	8	7	9	6	4	2
F25	2	5	6	9	7	8	3	4	1
F26	3	6	4	8	7	9	1	5	2
F27	2	6	4	9	7	8	3	5	1
F28	1	2	5	8	7	9	3	6	4
F29	2	6	4	9	7	8	1	5	3
F30	1	2	4	9	7	8	5	6	3
Sum of the ranks	51	131	131	256	207	230	89	130	80
Overall rank	1	5	5	8	6	7	3	4	2

### Solar PV parameter extraction

The proposed KGO framework is implemented for detailed parameter estimation and modelling of solar PV systems across SDM, DDM, TDM, and PV module structures. The objective function, described in “Mathematical model of solar PV system”, involves solving nonlinear equations; therefore, the NR method is integrated with the KGO algorithm to enhance the calculation process. The proposed KGO algorithm is applied to both a PV cell and a PV module, using experimental current–voltage data obtained from real measurements. For PV cell modelling, a 57 mm RTC France silicon solar cell tested under 1000 W/m² irradiance and 33 °C is considered. For PV module modelling, a Photowatt-PWP201 solar module tested under 1000 W/m² irradiance and 45 °C is used.

**Fig. 4:**
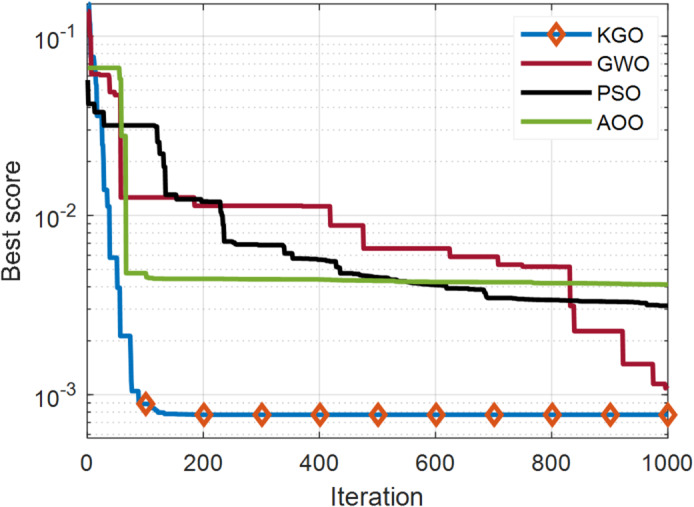
Convergence behaviour of the proposed KGO algorithm in compared with alternative algorithms for the SDM.

A. Analysis of SDM:

The KGO algorithm is first applied for parameter extraction of the SDM using practical experimental data. There are 26 operating current–voltage points available in this dataset for model validation. The estimated values of current and power, along with their absolute errors, are presented in Table 4. The maximum absolute current error is 1.362E−03, while the minimum is 1.652E−05, indicating that the proposed model closely matches real-world system behaviour and demonstrates high accuracy. In addition to accuracy, another important aspect of optimisation algorithms is their convergence rate. Therefore, a convergence comparison has been carried out to evaluate how quickly all three algorithms reach convergence. Since faster convergence is an essential requirement for optimization algorithms, this characteristic is evaluated through a comparative convergence analysis. A convergence plot of the KGO algorithm against well-established algorithms such as PSO, GWO, and AOO^36^ is presented in Fig. 4. The results clearly demonstrate that the proposed KGO algorithm achieves faster convergence while maintaining high accuracy.

The superior performance of KGO can be attributed to its unique mechanisms, including:

1. effective game-based strategies that achieves a good balance between exploration and exploitation,
2. the unique replacement mechanism for weaker candidates (players), and.
3. a nonlinear adaptive weight updating scheme that enhances convergence efficiency.

**Fig. 5:**
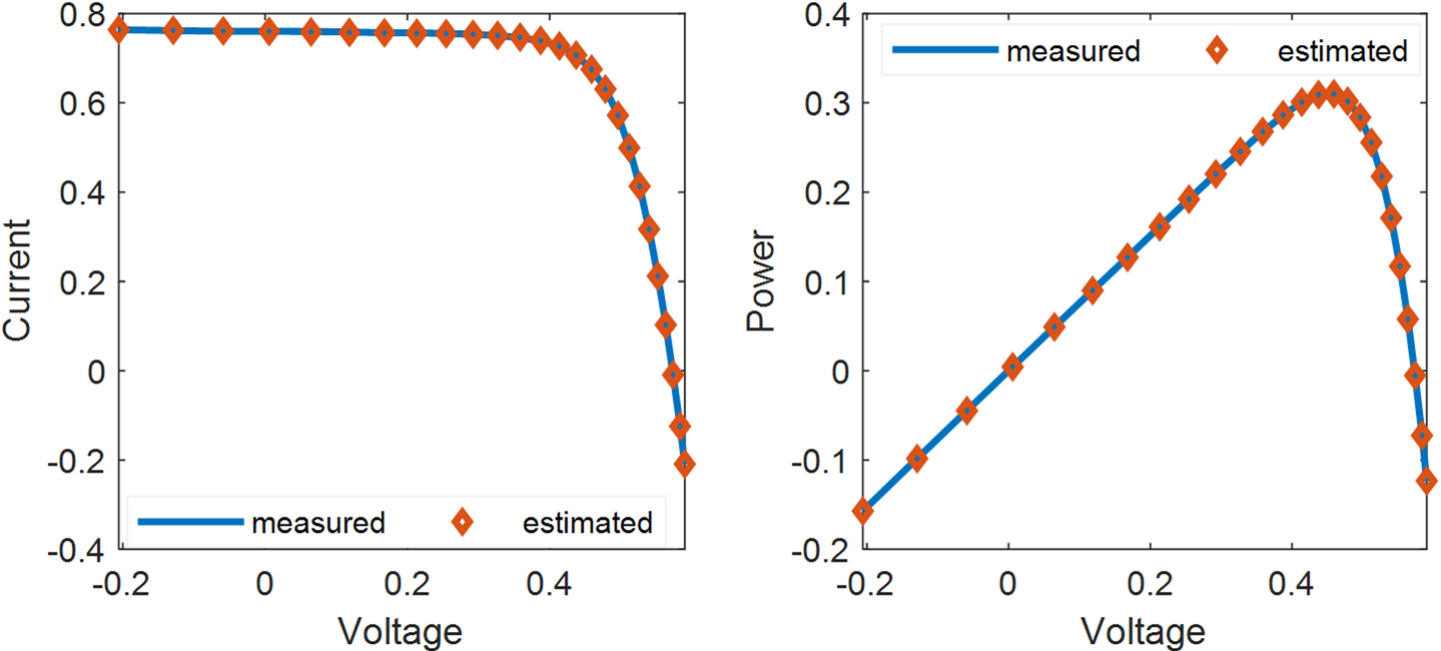
Comparison of experimental and KGO-estimated I–V and P–V characteristics of the solar PV system.

When applied to the SDM, the KGO algorithm achieves an objective function value of7.729857E−04. The corresponding I–V and P–V characteristics of the solar PV cell, plotted using the estimated data and compared with the experimental results, are illustrated in Fig. 5.

The estimated parameters obtained using the proposed KGO algorithm, along with those from recent algorithms, are presented in Table 5. The proposed algorithm outperforms all other methods primarily for two reasons. First, the computational efficiency of the proposed algorithm significantly enhances its performance. Second, the incorporation of the NR method within the optimization process improves accuracy, as the NR method effectively solves nonlinear equations and computes the estimated load current.

**Table 4:** Comparison of actual and KGO-estimated data points for the SDM.

S. no.	$\:{\boldsymbol{V}}_{\boldsymbol{L},\boldsymbol{m}\boldsymbol{e}\boldsymbol{s}}$	$\:{\boldsymbol{I}}_{\boldsymbol{L},\boldsymbol{m}\boldsymbol{e}\boldsymbol{s}}$	$\:{\boldsymbol{I}}_{\boldsymbol{L},\boldsymbol{c}\boldsymbol{a}\boldsymbol{l}\boldsymbol{c}}$	$\:{\boldsymbol{P}}_{\boldsymbol{L},\boldsymbol{c}\boldsymbol{a}\boldsymbol{l}\boldsymbol{c}}$	$\:{\boldsymbol{P}}_{\boldsymbol{L},\boldsymbol{c}\boldsymbol{a}\boldsymbol{l}\boldsymbol{c}}$	$\:|{\boldsymbol{I}}_{\boldsymbol{L},\boldsymbol{c}\boldsymbol{a}\boldsymbol{l}\boldsymbol{c}}\:-\:{\boldsymbol{I}}_{\boldsymbol{L},\boldsymbol{m}\boldsymbol{e}\boldsymbol{s}}|$	$\:\left|{\boldsymbol{P}}_{\boldsymbol{L},\boldsymbol{c}\boldsymbol{a}\boldsymbol{l}\boldsymbol{c}}-{\boldsymbol{P}}_{\boldsymbol{L},\boldsymbol{m}\boldsymbol{e}\boldsymbol{s}}\right|$
1	−0.2057	0.7640	0.7641	−0.1572	−0.1572	1.495E−04	3.074E−05
2	−0.1291	0.7620	0.7627	−0.0984	−0.0985	7.021E−04	9.065E−05
3	−0.0588	0.7605	0.7614	−0.0447	−0.0448	8.738E−04	5.138E−05
4	0.0057	0.7605	0.7602	0.0043	0.0043	3.455E−04	1.969E−06
5	0.0646	0.7600	0.7590	0.0491	0.0490	9.609E−04	6.208E−05
6	0.1185	0.7590	0.7580	0.0899	0.0898	9.892E−04	1.172E−04
7	0.1678	0.7570	0.7570	0.1270	0.1270	4.570E−05	7.669E−06
8	0.2132	0.7570	0.7561	0.1614	0.1612	9.152E−04	1.951E−04
9	0.2545	0.7555	0.7550	0.1923	0.1922	4.776E−04	1.216E−04
10	0.2924	0.7540	0.7536	0.2205	0.2204	4.026E−04	1.177E−04
11	0.3269	0.7505	0.7513	0.2453	0.2456	8.273E−04	2.704E−04
12	0.3585	0.7465	0.7473	0.2676	0.2679	8.053E−04	2.887E−04
13	0.3873	0.7385	0.7401	0.2860	0.2866	1.585E−03	6.137E−04
14	0.4137	0.7280	0.7274	0.3012	0.3009	5.738E−04	2.374E−04
15	0.4373	0.7065	0.7070	0.3090	0.3092	5.259E−04	2.300E−04
16	0.4590	0.6755	0.6754	0.3101	0.3100	9.974E−05	4.578E−05
17	0.4784	0.6320	0.6310	0.3023	0.3019	1.002E−03	4.793E−04
18	0.4960	0.5730	0.5722	0.2842	0.2838	8.253E−04	4.094E−04
19	0.5119	0.4990	0.4995	0.2554	0.2557	5.389E−04	2.759E−04
20	0.5265	0.4130	0.4135	0.2174	0.2177	4.848E−04	2.553E−04
21	0.5398	0.3165	0.3172	0.1708	0.1712	6.615E−04	3.571E−04
22	0.5521	0.2120	0.2120	0.1170	0.1171	1.652E−05	9.120E−06
23	0.5633	0.1035	0.1026	0.0583	0.0578	8.635E−04	4.864E−04
24	0.5736	−0.0100	−0.0093	−0.0057	−0.0053	7.013E−04	4.023E−04
25	0.5833	−0.1230	−0.1244	−0.0717	−0.0725	1.362E−03	7.942E−04
26	0.5900	−0.2100	−0.2091	−0.1239	−0.1234	8.977E−04	5.296E−04
$\:\sum\:_{i=1}^{N}\left|{I}_{L,calc}-{I}_{L,mes}\right|=1.7632E-02$				$\:\sum\:_{i=1}^{N}\left|{P}_{L,calc}-{P}_{L,mes}\right|=6.4806E-03$			

**Table 5:** Estimated parameters and objective function values for the SDM obtained using various algorithms from the literature.

Algorithm	$\:{\boldsymbol{I}}_{\boldsymbol{p}\boldsymbol{h}}\left(\boldsymbol{A}\right)$	$\:{\boldsymbol{I}}_{\boldsymbol{s}\boldsymbol{d}}\left(\boldsymbol{\mu\:}\boldsymbol{A}\right)$	$\:{\boldsymbol{R}}_{\boldsymbol{s}}$(Ω)	$\:{\boldsymbol{R}}_{\boldsymbol{s}\boldsymbol{h}}\left(\boldsymbol{\Omega\:}\right)$	$\:\boldsymbol{n}$	F
KGO	0.760788	0.31069	0.036547	52.88991	1.477272	7.729857E−04
iAPO^37^	0.7608	0.3230	0.0364	53.7185	1.4812	9.8602E−04
APO^37^	0.7608	0.3230	0.0364	53.7185	1.4812	9.8602E−04
Q-Jaya^38^	0.7607	0.3230	0.3230	53.7185	1.4812	9.86E − 04
E-JAYA^39^	0.7608	0.3230	0.0364	53.7185	1.4812	9.860E−04
DNMRIME^40^	0.7608	0.3230	0.0364	53.7190	1.4812	9.8602E−04
RIME^40^	0.7610	0.3339	3.6247	52.0251	1.4846	1.002E−03
TSIA^41^	0.7606	0.3298	0.0363	56.5694	1.4832	9.9339E − 04
FVIM-DE^42^	0.7608	0.3230	0.0364	53.7185	1.4811	9.8602E−04
I-JAYA^43^	0.7608	0.3228	0.0364	53.7595	1.4811	9.860E−04
VABES^44^	0.7608	0.0323	0.0364	53.7185	1.4811	9.8602E−04
I-CPA^45^	0.7606	0.3256	0.0364	56.8036	1.4819	9.9862E − 04
FIPSO-SQP^46^	0.7608	0.3226	0.0363	53.6800	1.4810	9.86E − 04
GO-TLBO^15^	0.7608	0.3316	0.0363	54.1154	1.4838	9.874E−04
PIFN^47^	0.7608	0.5480	0.0338	63.9870	1.5360	7.72E−04
ISSO^48^	NA	NA	NA	NA	NA	9.860E−04
BEA^49^	0.7607	0.3230	0.0364	53.7180	1.4810	9:86E − 04
CLPSO^50^	NA	NA	NA	NA	NA	9.963E−04
SMA^51^	0.7608	0.3231	0.0364	53.7149	1.4811	9.801E−04
DSCSE^52^	0.7608	0.0000	0.0364	53.7185	1.4812	9.860E−04
MSA^53^	0.7608	0.3000	0.0364	53.7185	1.4812	9.86E − 04
WLCSODGM^54^	0.7608	0.3230	0.0364	53.7185	1.4812	9.860E−04
MS-TSA^55^	0.7608	0.3204	0.0364	53.4685	1.4803	9.8642E−04
WHHO^56^	0.7608	0.3230	0.0364	53.7187	1.4811	9.860E−04
EMNOA^57^	0.7808	0.3230	0.0364	53.7185	1.4811	9.8602E−04
DMO^58^	0.7608	0.3230	0.0364	53.7190	1.4811	9.8602E − 04
EO-TLBO^59^	0.7608	0.3230	0.0364	53.7185	1.4812	9.860E−04
IWOA^60^	0.7608	0.3232	0.0364	53.7317	1.4812	9.860E−04
LCROA^61^	0.7608	0.3107	0.0366	52.8898	1.5169	7.730E−04
IMP^62^	0.7608	0.3230	0.0364	53.7185	1.4812	9.860E−04
GSKA^63^	0.7608	0.3231	0.3231	53.7220	1.4810	9.86E − 04

**Fig. 6:**
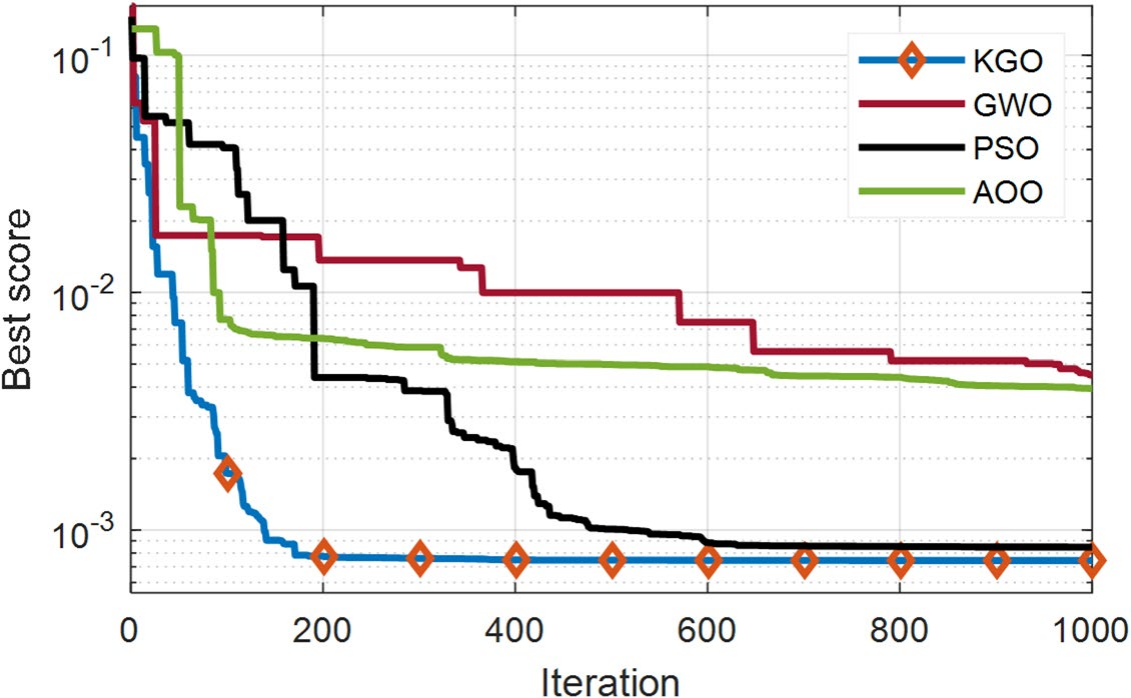
Convergence trends of the proposed KGO algorithm compared with other established algorithms for the DDM.

B. Analysis for DDM:

The degree of nonlinearity for finding the objective function is higher in the case of the DDM compared to the SDM. This is because there are seven unknown parameters to be estimated with more complex equations. The effectiveness of the proposed KGO algorithm, in terms of both accuracy and faster convergence, is evident from the convergence curves illustrated in Fig. 6. The KGO algorithm outperforms the compared methods by converging within 200 iterations and reaches the final value. In contrast, the other algorithms fail to achieve convergence. When applied to the DDM, the KGO algorithm achieves an objective function value of 7.43146E−04. Table 6 presents the real and estimated data points along with the absolute current and power errors. The maximum and minimum absolute current errors are 1.0213E−03 and 2.7385E−05, respectively, indicating that the mathematical model closely approximates the practical system. Table 7 presents the estimated parameters for the DDM along with the RMSE value, compared with its peers. The proposed KGO algorithm ranks first in terms of accuracy.

**Table 6:** Comparison of actual and KGO-predicted data points for the DDM.

S.No	$\:{\boldsymbol{V}}_{\boldsymbol{L},\boldsymbol{m}\boldsymbol{e}\boldsymbol{s}}$	$\:{\boldsymbol{I}}_{\boldsymbol{L},\boldsymbol{m}\boldsymbol{e}\boldsymbol{s}}$	$\:{\boldsymbol{I}}_{\boldsymbol{L},\boldsymbol{c}\boldsymbol{a}\boldsymbol{l}\boldsymbol{c}}$	$\:{\boldsymbol{P}}_{\boldsymbol{L},\boldsymbol{m}\boldsymbol{e}\boldsymbol{s}}$	$\:{\boldsymbol{P}}_{\boldsymbol{L},\boldsymbol{c}\boldsymbol{a}\boldsymbol{l}\boldsymbol{c}}$	$\:|{\boldsymbol{I}}_{\boldsymbol{L},\boldsymbol{c}\boldsymbol{a}\boldsymbol{l}\boldsymbol{c}}\:-\:{\boldsymbol{I}}_{\boldsymbol{L},\boldsymbol{m}\boldsymbol{e}\boldsymbol{s}}|$	$\:\left|{\boldsymbol{P}}_{\boldsymbol{L},\boldsymbol{c}\boldsymbol{a}\boldsymbol{l}\boldsymbol{c}}-{\boldsymbol{P}}_{\boldsymbol{L},\boldsymbol{m}\boldsymbol{e}\boldsymbol{s}}\right|$
1	−0.2057	0.7640	0.7640	−0.1572	−0.1571	2.7385E−05	5.6332E−06
2	−0.1291	0.7620	0.7626	−0.0984	−0.0985	6.0046E−04	7.7519E−05
3	−0.0588	0.7605	0.7613	−0.0447	−0.0448	8.4080E−04	4.9439E−05
4	0.0057	0.7605	0.7602	0.0043	0.0043	3.1622E−04	1.8025E−06
5	0.0646	0.7600	0.7591	0.0491	0.0490	8.7699E−04	5.6654E−05
6	0.1185	0.7590	0.7581	0.0899	0.0898	8.6034E−04	1.0195E−04
7	0.1678	0.7570	0.7572	0.1270	0.1271	2.0529E−04	3.4448E−05
8	0.2132	0.7570	0.7563	0.1614	0.1612	7.4618E−04	1.5908E−04
9	0.2545	0.7555	0.7552	0.1923	0.1922	3.2902E−04	8.3735E−05
10	0.2924	0.7540	0.7537	0.2205	0.2204	3.1029E−04	9.0727E−05
11	0.3269	0.7505	0.7513	0.2453	0.2456	8.3012E−04	2.7137E−04
12	0.3585	0.7465	0.7472	0.2676	0.2679	7.0147E−04	2.5148E−04
13	0.3873	0.7385	0.7399	0.2860	0.2866	1.3895E−03	5.3815E−04
14	0.4137	0.7280	0.7272	0.3012	0.3008	8.0549E−04	3.3323E−04
15	0.4373	0.7065	0.7068	0.3090	0.3091	3.3815E−04	1.4787E−04
16	0.4590	0.6755	0.6753	0.3101	0.3100	1.7183E−04	7.8868E−05
17	0.4784	0.6320	0.6311	0.3023	0.3019	9.3807E−04	4.4877E−04
18	0.4960	0.5730	0.5723	0.2842	0.2839	6.6646E−04	3.3056E−04
19	0.5119	0.4990	0.4997	0.2554	0.2558	7.1167E−04	3.6430E−04
20	0.5265	0.4130	0.4136	0.2174	0.2178	5.9135E−04	3.1135E−04
21	0.5398	0.3165	0.3172	0.1708	0.1712	6.5825E−04	3.5533E−04
22	0.5521	0.2120	0.2119	0.1170	0.1170	9.0634E−05	5.0039E−05
23	0.5633	0.1035	0.1025	0.0583	0.0577	1.0213E−03	5.7532E−04
24	0.5736	−0.0100	−0.0094	−0.0057	−0.0054	5.7486E−04	3.2974E−04
25	0.5833	−0.1230	−0.1244	−0.0717	−0.0725	1.3561E−03	7.9103E−04
26	0.5900	−0.2100	−0.2089	−0.1239	−0.1233	1.0672E−03	6.2964E−04
$\:\sum\:_{i=1}^{N}\left|{I}_{L,calc}-{I}_{L,mes}\right|=1.7025E-02$				$\:\sum\:_{i=1}^{N}\left|{P}_{L,calc}-{P}_{L,mes}\right|=6.4680E-03$			

**Table 7:** Estimated parameters and objective function values for the DDM obtained using different algorithms from the literature.

Algorithm	$\:{\boldsymbol{I}}_{\boldsymbol{p}\boldsymbol{h}}$(A)	$\:{\boldsymbol{I}}_{\boldsymbol{s}\boldsymbol{d}1}\left(\mu\:A\right)$	$\:{\boldsymbol{I}}_{\boldsymbol{s}\boldsymbol{d}2}$($\:\mu\:A)$	$\:{\boldsymbol{R}}_{\boldsymbol{s}}$(Ω)	$\:{\boldsymbol{R}}_{\boldsymbol{s}\boldsymbol{h}}$(Ω)	$\:\boldsymbol{n}1$	$\:\boldsymbol{n}2$	F
KGO	0.7608	0.1207	0.9999	0.0374	55.7915	1.4019	1.8720	7.431462E−04
EOTLBO^59^	0.7610	0.2260	0.7490	0.0367	55.5000	1.4500	2.0000	9.8200E−04
iAPO^37^	0.7608	0.2259	0.0367	1.4510	55.4854	0.7493	2.0000	9.8248E−04
APO^37^	0.7608	0.2179	0.0368	1.4480	55.6288	0.8169	2.000	9.8252E−04
WLCSODGM^54^	0.7608	0.7492	0.2260	0.0367	55.4850	2.0000	1.4510	9.8248E−04
DNMRIME^40^	0.7608	0.2259	0.7496	0.0367	55.4860	1.4510	2.0000	9.8248E*04
RIME^40^	0.7610	0.2944	0.4025	0.0360	58.2079	1.4760	1.9300	1.0243E−03
IWOA^60^	0.7608	0.6770	0.2350	0.0360	55.4080	2.0000	1.4540	9.8300E − 04
FVIM-DE^42^	0.7608	0.2260	0.7493	0.0037	55.4854	1.4510	2.0000	9.8248E−04
IMP^62^	0.7608	0.2260	0.7493	0.0367	55.4854	1.4510	2.0000	9.8249E−04
MS-TSA^55^	0.7608	0.2626	0.4628	0.0365	54.6944	1.4637	1.9997	9.8356E−04
VABES^44^	0.7608	0.2670	0.4070	0.0366	54.6625	1.4647	1.9967	9.8332E−04
SMA^51^	0.7608	0.7487	0.2265	0.0368	55.7146	2.0000	1.4546	9.8149E−04
I-CPA^45^	0.7602	0.0404	0.2869	0.0365	63.0435	1.5227	1.4779	1.0200E − 03
PIFN^47^	0.7604	0.2750	0.3745	0.0352	63.7688	1.9427	1.4989	7.5900E−04
GOTLBO^15^	0.7608	0.8002	0.2205	0.0368	56.0753	1.9997	1.4490	9.8742E−04
TSIA^41^	0.7609	0.2166	0.2674	0.0367	52.5696	1.4503	1.7552	9.8800E − 04
DSCSE^52^	0.7608	0.2167	0.0000	0.0367	55.3750	2.0000	1.4532	9.8251E−04
EMNOA^57^	0.7608	0.2260	0.7493	0.0367	55.4854	1.4510	2.0000	9.8603E−04
EJAYA^39^	0.7608	0.2260	0.7493	0.0367	55.4851	1.4510	2.0000	9.8248E−04
R-TLBO^64^	0.7608	0.2290	0.8500	0.0363	49.0850	1.4550	1.9610	9.800E − 04
WHHO^56^	0.7608	0.2286	0.7272	0.0367	55.4264	1.4519	2.0000	9.8249E−04
MSA^53^	0.7608	0.2430	0.6046	0.0367	55.1200	1.4571	1.9969	9.8270E − 04
IJAYA^43^	0.7601	0.0050	0.7509	0.0376	77.8519	1.2186	1.6247	9.8293E−04
DMO^58^	0.7608	0.4278	0.2633	0.0366	54.7040	1.9919	1.4638	9.832E − 04
LCROA^61^	0.7610	0.1460	0.7350	0.0372	54.9000	1.4600	1.8900	7.4900E−04
IWOA^60^	0.7608	0.6771	0.2355	0.0367	55.4082	2.0000	1.4545	9.8255E−04
ISSO^48^	NA	NA	NA	NA	NA	NA	NA	9.8248E−04

A Friedman rank analysis was conducted on algorithms common to both SDM and DDM models. The Friedman test indicates significant differences among algorithms (*p* < 0.05) as presented in Table 8. KGO obtains the lowest average rank across SDM and DDM, followed by LCROA, confirming its superior and consistent performance.

**Table 8:** Friedman ranking of algorithms across SDM and DDM PV models.

Algorithm	SDM rank	DDM rank	Overall rank
KGO	1.5	1	1.25
LCROA	1.5	2	1.75
DNMRIME	11	9	10
FVIM-DE	11	9	10
E-JAYA	11	9	10
EMNOA	11	9	10
IWOA	11	9	10
BLPSO	11	9	10
I-JAYA	11	9	10
IMP	11	9	10
WHHO	11	9	10
WLCSODGM	11	9	10
MS-TSA	11	9	10
SMA	3	17	10
GO-TLBO	15	9	12
CLPSO	17	9	13
VABES	11	16	13.5
RIME	18	15	16.5

**Fig. 7:**
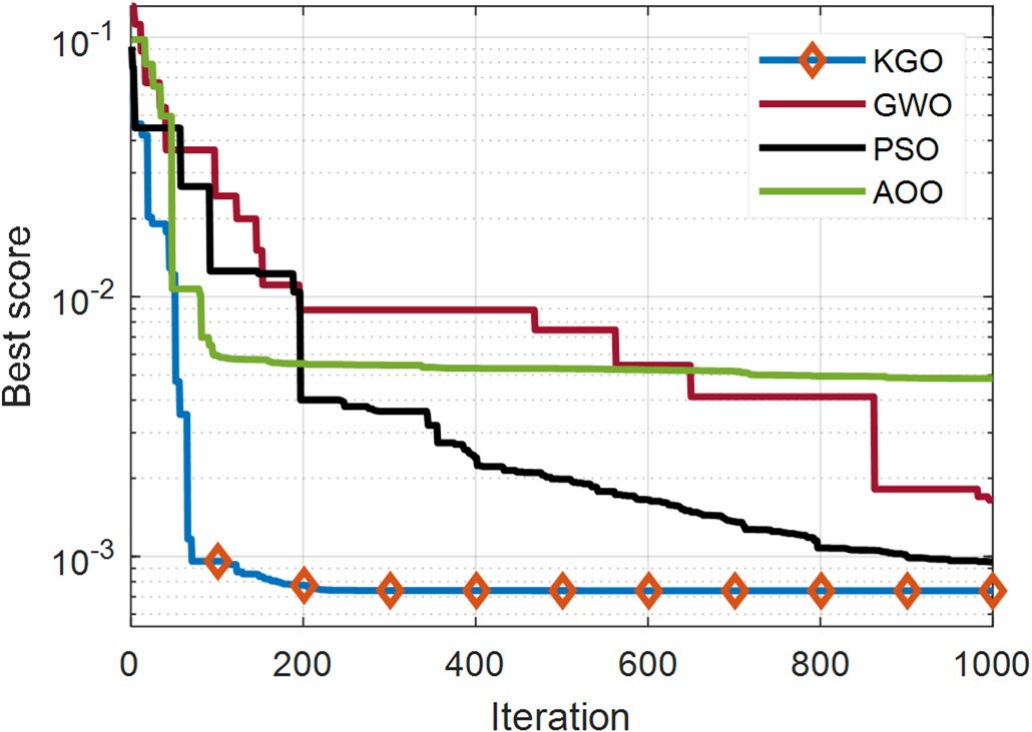
Convergence behaviour of the proposed KGO algorithm versus other benchmark algorithms for the TDM.

C. Analysis for TDM:

Modelling a solar PV cell using the TDM approach is more complex because it involves nine unknown parameters and a high degree of nonlinearity. Table 9 presents both the measured and estimated current values, together with the corresponding absolute errors in current and power. For the TDM model, the objective function value is 7.3771E−04, which is notably lower than those of the SDM and DDM models. Figure 7 illustrates the convergence curves of the proposed KGO algorithm with other algorithms. The results demonstrate that KGO achieves faster convergence with greater efficiency than the compared algorithms.

**Table 9:** Comparison of actual and KGO-estimated data points for the TDM.

S.No	$\:{\boldsymbol{V}}_{\boldsymbol{L},\boldsymbol{m}\boldsymbol{e}\boldsymbol{s}}$	$\:{\boldsymbol{I}}_{\boldsymbol{L},\boldsymbol{m}\boldsymbol{e}\boldsymbol{s}}$	$\:{\boldsymbol{I}}_{\boldsymbol{L},\boldsymbol{c}\boldsymbol{a}\boldsymbol{l}\boldsymbol{c}}$	$\:{\boldsymbol{P}}_{\boldsymbol{L},\boldsymbol{m}\boldsymbol{e}\boldsymbol{s}}$	$\:{\boldsymbol{P}}_{\boldsymbol{L},\boldsymbol{c}\boldsymbol{a}\boldsymbol{l}\boldsymbol{c}}$	$\:|{\boldsymbol{I}}_{\boldsymbol{L},\boldsymbol{c}\boldsymbol{a}\boldsymbol{l}\boldsymbol{c}}\:-\:{\boldsymbol{I}}_{\boldsymbol{L},\boldsymbol{m}\boldsymbol{e}\boldsymbol{s}}|$	$\:\left|{\boldsymbol{P}}_{\boldsymbol{L},\boldsymbol{c}\boldsymbol{a}\boldsymbol{l}\boldsymbol{c}}-{\boldsymbol{P}}_{\boldsymbol{L},\boldsymbol{m}\boldsymbol{e}\boldsymbol{s}}\right|$
1	−0.2057	0.764	0.7638	−0.1572	−0.1571	1.5172E−04	3.1208E−05
2	−0.1291	0.762	0.7625	−0.0984	−0.0984	5.3121E−04	6.8579E−05
3	−0.0588	0.7605	0.7613	−0.0447	−0.0448	8.2191E−04	4.8329E−05
4	0.0057	0.7605	0.7602	0.0043	0.0043	2.8947E−04	1.6500E−06
5	0.0646	0.76	0.7592	0.0491	0.0490	8.1012E−04	5.2334E−05
6	0.1185	0.759	0.7582	0.0899	0.0899	7.6036E−04	9.0103E−05
7	0.1678	0.757	0.7573	0.1270	0.1271	3.2802E−04	5.5042E−05
8	0.2132	0.757	0.7564	0.1614	0.1613	6.1628E−04	1.3139E−04
9	0.2545	0.7555	0.7553	0.1923	0.1922	2.1423E−04	5.4521E−05
10	0.2924	0.754	0.7538	0.2205	0.2204	2.3821E−04	6.9653E−05
11	0.3269	0.7505	0.7513	0.2453	0.2456	8.3295E−04	2.7229E−04
12	0.3585	0.7465	0.7471	0.2676	0.2678	6.2010E−04	2.2231E−04
13	0.3873	0.7385	0.7397	0.2860	0.2865	1.2346E−03	4.7817E−04
14	0.4137	0.728	0.7270	0.3012	0.3008	9.9055E−04	4.0979E−04
15	0.4373	0.7065	0.7067	0.3090	0.3090	1.8922E−04	8.2745E−05
16	0.459	0.6755	0.6753	0.3101	0.3100	2.2351E−04	1.0259E−04
17	0.4784	0.632	0.6311	0.3023	0.3019	8.7406E−04	4.1815E−04
18	0.496	0.573	0.5725	0.2842	0.2839	5.2127E−04	2.5855E−04
19	0.5119	0.499	0.4999	0.2554	0.2559	8.6750E−04	4.4407E−04
20	0.5265	0.413	0.4137	0.2174	0.2178	6.8753E−04	3.6198E−04
21	0.5398	0.3165	0.3172	0.1708	0.1712	6.5650E−04	3.5438E−04
22	0.5521	0.212	0.2118	0.1170	0.1169	1.8500E−04	1.0214E−04
23	0.5633	0.1035	0.1023	0.0583	0.0576	1.1612E−03	6.5410E−04
24	0.5736	−0.01	−0.0095	−0.0057	−0.0055	4.6183E−04	2.6491E−04
25	0.5833	−0.123	−0.1244	−0.0717	−0.0725	1.3543E−03	7.8994E−04
26	0.59	−0.21	−0.2088	−0.1239	−0.1232	1.2120E−03	7.1511E−04
$\:\sum\:_{i=1}^{N}\left|{I}_{L,calc}-{I}_{L,mes}\right|=1.6834E-02$				$\:\sum\:_{i=1}^{N}\left|{P}_{L,calc}-{P}_{L,mes}\right|=6.5340E-03$			

**Fig. 8:**
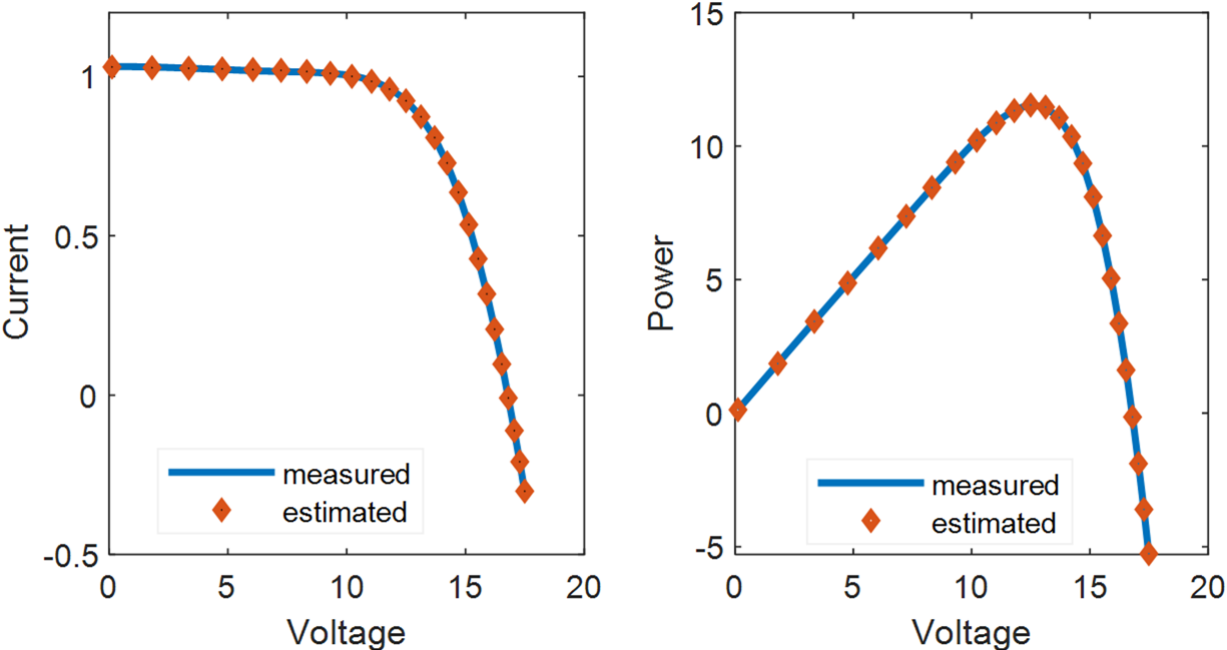
I-V and P-V curves using the real and estimated values for PWP-201 PV module.

**Fig. 9:**
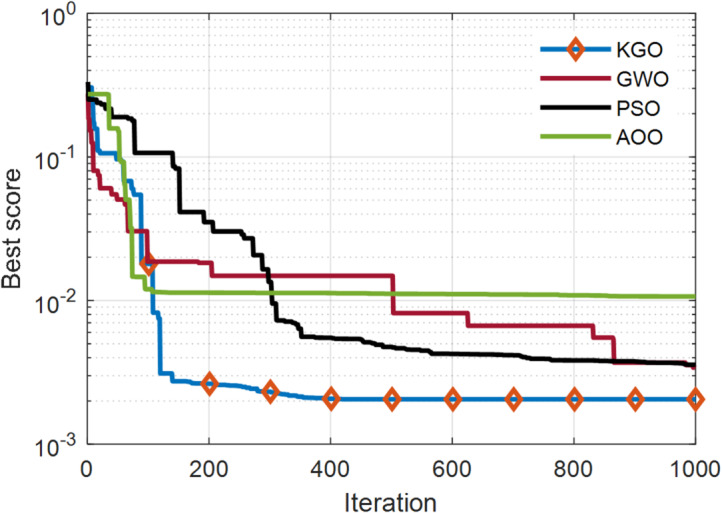
Convergence behaviour of the proposed KGO algorithm versus other benchmark algorithms for the PWP-201 PV Module.

D. Analysis of PWP-201 PV Module:

Table 10 summarizes the estimated current () and power () values, along with the corresponding absolute errors in current and power. When compared with well-known algorithms reported in the solar cell literature, the KGO algorithm exhibits superior performance by achieving a lower objective function value of 2.0529E−03. The estimated I–V and P–V characteristics obtained using the KGO algorithm, as illustrated in Fig. 8, show a close resemblance to the experimental measurements. In addition, Fig. 9 presents the convergence behaviour of the KGO algorithm in comparison with other algorithms, demonstrating its faster convergence. These results confirm the high accuracy of solar PV modelling using the KGO algorithm. In addition, Table 11 compares the parameter estimates and objective function values obtained using the KGO algorithm with those produced by the other three algorithms for the same PV modules, further demonstrating the effectiveness and robustness of the proposed approach.

**Table 10:** Comparison of measured and KGO-estimated data points for the PV module.

S.No	$\:{\boldsymbol{V}}_{\boldsymbol{L},\boldsymbol{m}\boldsymbol{e}\boldsymbol{s}}$	$\:{\boldsymbol{I}}_{\boldsymbol{L},\boldsymbol{m}\boldsymbol{e}\boldsymbol{s}}$	$\:{\boldsymbol{I}}_{\boldsymbol{L},\boldsymbol{c}\boldsymbol{a}\boldsymbol{l}\boldsymbol{c}}$	$\:{\boldsymbol{P}}_{\boldsymbol{L},\boldsymbol{m}\boldsymbol{e}\boldsymbol{s}}$	$\:{\boldsymbol{P}}_{\boldsymbol{L},\boldsymbol{c}\boldsymbol{a}\boldsymbol{l}\boldsymbol{c}}$	$\:|{\boldsymbol{I}}_{\boldsymbol{L},\boldsymbol{c}\boldsymbol{a}\boldsymbol{l}\boldsymbol{c}}\:-\:{\boldsymbol{I}}_{\boldsymbol{L},\boldsymbol{m}\boldsymbol{e}\boldsymbol{s}}|$	$\:\left|{\boldsymbol{P}}_{\boldsymbol{L},\boldsymbol{c}\boldsymbol{a}\boldsymbol{l}\boldsymbol{c}}-{\boldsymbol{P}}_{\boldsymbol{L},\boldsymbol{m}\boldsymbol{e}\boldsymbol{s}}\right|$
1	0.1248	1.0315	1.0297	0.1287	0.1285	1.7717E−03	2.2111E−04
2	1.8093	1.03	1.0277	1.8636	1.8593	2.3390E−03	4.2319E−03
3	3.3511	1.026	1.0257	3.4382	3.4373	2.7549E−04	9.2318E−04
4	4.7622	1.022	1.0238	4.8670	4.8757	1.8334E−03	8.7309E−03
5	6.0538	1.018	1.0218	6.1628	6.1859	3.8137E−03	2.3087E−02
6	7.2364	1.0155	1.0193	7.3486	7.3762	3.8231E−03	2.7665E−02
7	8.3189	1.014	1.0157	8.4354	8.4498	1.7334E−03	1.4420E−02
8	9.3097	1.01	1.0100	9.4028	9.4026	2.1350E−05	1.9876E−04
9	10.2163	1.0035	1.0004	10.2521	10.2205	3.0905E−03	3.1574E−02
10	11.0449	0.988	0.9847	10.9124	10.8764	3.2573E−03	3.5976E−02
11	11.8018	0.963	0.9602	11.3651	11.3320	2.8113E−03	3.3179E−02
12	12.4929	0.9255	0.9239	11.5622	11.5419	1.6262E−03	2.0316E−02
13	13.1231	0.8725	0.8736	11.4499	11.4639	1.0667E−03	1.3998E−02
14	13.6983	0.8075	0.8082	11.0614	11.0710	7.0315E−04	9.6319E−03
15	14.2221	0.7265	0.7286	10.3324	10.3615	2.0502E−03	2.9158E−02
16	14.6995	0.6345	0.6366	9.3268	9.3582	2.1336E−03	3.1362E−02
17	15.1346	0.5345	0.5354	8.0894	8.1035	9.2821E−04	1.4048E−02
18	15.5311	0.4275	0.4282	6.6395	6.6504	6.9954E−04	1.0865E−02
19	15.8929	0.3185	0.3179	5.0619	5.0516	6.4989E−04	1.0329E−02
20	16.2229	0.2085	0.2070	3.3825	3.3583	1.4917E−03	2.4200E−02
21	16.5241	0.101	0.0976	1.6689	1.6135	3.3554E−03	5.5446E−02
22	16.7987	−0.008	−0.0086	−0.1344	−0.1443	5.8898E−04	9.8940E−03
23	17.0499	−0.111	−0.1110	−1.8925	−1.8921	2.7994E−05	4.7730E−04
24	17.2793	−0.209	−0.2086	−3.6114	−3.6046	3.9294E−04	6.7897E−03
25	17.4885	−0.303	−0.3009	−5.2990	−5.2628	2.0730E−03	3.6254E−02
$\:\sum\:_{i=1}^{N}\left|{I}_{L,calc}-{I}_{L,mes}\right|=4.2558E-02$				$\:\sum\:_{i=1}^{N}\left|{P}_{L,calc}-{P}_{L,mes}\right|=4.5298E-01$			

**Table 11:** Estimated parameters and corresponding objective function values of the PV module obtained using different algorithms reported in the literature.

Algorithm	$\:{\boldsymbol{I}}_{\boldsymbol{p}\boldsymbol{h}}$(A)	$\:{\boldsymbol{I}}_{\boldsymbol{s}\boldsymbol{d}}$ ($\:\mu\:A)$	$\:{\boldsymbol{R}}_{\boldsymbol{s}}$ (Ω)	$\:{\boldsymbol{R}}_{\boldsymbol{s}\boldsymbol{h}}$ (Ω)	$\:\boldsymbol{n}$	F
KGO	1.0314	2.6381	0.0343	22.8233	1.2977	2.05296E−03
CMMDE^51^	1.0305	3.4823	1.2013	981.9823	48.6428	2.4251E−03
Rao^59^	1.0343	1.3727	1.3042	554.5480	47.0848	2.8220E−03
DNMRIME^40^	1.0305	3.4823	1.2012	982.0087	48.6429	2.4251E−03
RIME^40^	1.0310	3.7774	1.1917	978.2530	48.9589	2.4505E−03
LETLBO^43^	1.0306	3.4705	1.2015	974.6190	48.6301	2.4251E−03
FVIM-DE^42^	1.0305	0.3482	0.0334	27.2773	1.3512	2.4251E−03
E-JAYA^39^	1.0305	3.4823	1.2013	981.9824	48.6428	2.4251E−03
SA^51^	1.0331	3.6642	1.1989	833.3333	48.8211	2.7000E−03
GO-TLBO^15^	1.0305	3.4823	1.2013	981.9776	48.6428	2.4251E−03
CARO^65^	1.0319	3.2840	1.2056	841.3213	48.4036	2.4270E−03
VABES^44^	1.0396	0.1320	0.8683	699.1798	1.3491	2.5400E−03
MLBSA^39^	1.0305	3.4823	1.2013	981.9822	48.6428	2.4251E − 03
AGDE^43^	1.0305	3.4823	1.2013	981.9822	48.6428	2.4251E − 03
ABC^59^	1.0335	3.6785	1.2133	559.1100	48.8326	2.9946E−03
EMNOA^57^	1.0305	3.4823	0.0334	27.2773	1.3512	2.4251E−03
BLPSO^51^	1.0305	3.5176	1.2002	992.7901	48.6815	2.4252E−03
STLBO^56^	1.0305	3.4824	1.2013	982.0387	1.3511	2.4251E−03
CMAE^43^	1.0380	3.7941	1.1822	693.7804	48.9761	3.9926E−03
LBSA^51^	1.0305	3.4901	1.2010	987.7807	48.6513	2.4252E−03
SMA^59^	1.0342	1.3214	1.2564	559.4500	45.1993	2.8113E−03
BLPSO^56^	1.0305	3.5176	1.2002	992.7901	1.3522	2.4252E−03
IMP^62^	NA	NA	NA	NA	NA	2.4251E−03
CS^59^	1.0354	2.3075	1.2364	551.1100	48.9746	2.8257E−03
IWOA^60^	1.0305	3.4717	1.2016	978.6771	48.6313	2.4251E−03
RcrIJADE^19^	1.0305	3.4823	1.2013	981.9822	48.6428	2.4251E−03
EHHO^56^	1.0306	3.4600	1.2019	971.2760	1.3493	2.4252E−03
CLPSO^50^	1.0304	3.6131	1.1978	1017.0000	48.7847	2.4281E−03
I-JAYA^43^	1.0305	3.4840	1.2013	983.9256	48.6447	2.4251E−03
LCROA^61^	NA	NA	NA	NA	NA	2.0954E−03
WHHO^56^	1.0305	3.4821	1.2013	981.9052	1.3500	2.4250E−03
WLCSODGM^54^	1.0305	3.4823	1.2013	981.9823	48.6428	2.4251E−03
MS-TSA^55^	1.0305	3.500	1.201	987.5209	48.6624	2.4251E−03

E. Convergence and stability analysis:

To further validate the robustness and reliability of the proposed KGO algorithm a comprehensive convergence and stability analysis was conducted^66,67^. Each of the PV models (SDM, DDM, TDM, and PV Module) had 30 independent optimization runs processed similarly but used random initialization each time.

Table 12 summarizes the mean, median, and standard deviation of the RMSE obtained over these independent runs. The similarity of the average and median value for all of the models indicates that the results were not impacted by outlier RMSE values. Additionally, the very low amount of variability across these independent runs shows how very consistently and reliably the proposed methodology produces results.

Moreover, in combination with the convergence curves shown in Figs. 4, 6 and 7, and 9, the statistical results in Table 12 verify that KGO converges rapidly toward the global optimum and maintains stable performance across repeated runs. These findings confirm that the reported solutions are not incidental outcomes of stochastic behavior but represent reliable and statistically stable optima, thereby satisfying the requirements for rigorous convergence and stability analysis.

**Table 12:** Convergence and stability analysis of KGO over 30 independent runs for different PV models.

PV model	Mean	Median	STD
SDM	7.73E−04	7.73E−04	2.37E-17
DDM	7.45E−04	7.43E−04	6.02E−08
TDM	7.40E−04	7.39E−04	8.65E−04
PV Module	2.06E−03	2.05E−04	4.19E−04

For each PV model (SDM, DDM, TDM), Wilcoxon signed-rank tests were performed between KGO and PSO, GWO and AOO using RMSE values from 30 independent paired runs and the results are presented in Table 13. In all pairwise comparisons, the obtained p-values were lower than 0.05, indicating that the null hypothesis of equal performance can be rejected at the 95% confidence level. Therefore, KGO is statistically superior to PSO, GWO and AOO for all models.

**Table 13:** Wilcoxon signed-rank test results comparing the proposed KGO with PSO, GWO, and AOO based on RMSE values obtained from 30 independent paired runs for SDM, DDM, TDM.

Model	Pair	*p*-value	Decision
SDM	KGO vs. PSO	1.95E−03	Reject H_0_
SDM	KGO vs. GWO	1.95E−03	Reject H_0_
SDM	KGO vs. AOO	1.95E−03	Reject H_0_
DDM	KGO vs. PSO	1.95E−02	Reject H_0_
DDM	KGO vs. GWO	1.95E−03	Reject H_0_
DDM	KGO vs. AOO	1.95E−03	Reject H_0_
TDM	KGO vs. PSO	1.60E−01	Reject H_0_
TDM	KGO vs. GWO	1.95E−03	Reject H_0_
TDM	KGO vs. AOO	1.95E−03	Reject H_0_

## Conclusions

In this work, a novel metaheuristic algorithm, the Kabaddi Game Optimizer, inspired by the strategic elements of the traditional South Asian sport Kabaddi, has been proposed and validated. The algorithm incorporates two game-inspired strategies (Dubki and Akraman), an adaptive weight updating mechanism, and a weak-player replacement process, enabling a balance between exploration and exploitation. The performance of KGO was first benchmarked using the CEC 2017 test suite, where it consistently outperformed seven well-established algorithms, securing the top rank. Subsequently, the algorithm was applied to the parameter estimation problem of solar PV modelling. KGO successfully modelled three standard PV cell models (SDM, DDM, TDM) and a PV module (PWP-201), achieving low RMSE values of 7.729857E−04, 7.43146E−04, 7.3771E−04, and 2.0529E−03, respectively. The results presented show greater accuracy, faster convergence, and superior robustness when using KGO versus current methods available on the market.

While the results are promising, several opportunities exist for future research. First, the proposed KGO can be extended to multi-objective optimization problems, where trade-offs between conflicting objectives such as accuracy, computational cost, and robustness must be addressed. Furthermore, KGO has potential to be combined with traditional optimization approaches such as Newton-Raphson and Gradient Based methods. This hybridisation could be beneficial in increasing convergence speeds for complex case studies. Additionally, applying KGO to broader domains, including renewable energy forecasting, power system optimization, image processing, and machine learning model training, could highlight its adaptability and generalizability.

## Data Availability

The datasets used and/or analyzed during the current study are available from the corresponding author on reasonable request.

## List of symbols

$\:{I}_{sh}$: Shunt resistance current
$$\:{I}_{d}$$: Diode current
$$\:{I}_{ph}$$: Photocurrent
$$\:{I}_{L}$$: Output current
$\:{I}_{sd}$: Diode reverse saturation current
$\:n$: Diode ideality factor
$$\:q$$: Electron charge
$$\:k$$: Boltzmann constant
$\:{V}_{L}$: PV cell output voltage
$\:N$: Number of data points
RMSE: Root-Mean square error
$$\:{I}_{L.mes}$$: Measured current
$$\:{R}_{sh}$$: Shunt resistance
$$\:{F}_{obj}$$: Objective function
$\:{N}_{p}\:$: Number of cells in parallel
$$\:{I}_{L.calc}$$: Estimated current
$\:{N}_{s}\:$: Number of cells in series
$$\:T$$: Temperature
$$\:f\left(x\right)$$: Nonlinear function
SDM: Single diode model
PSO: Particle Swarm Optimization
NR: Newton-Raphson
$\:{f}^{{\prime\:}\left(x\right)}$: Derivative of nonlinear function
DDM: Double Diode model
PV: Photovoltaic
DE: Differential Evolution
TDM: Triple diode model
$\:\varDelta\:{V}_{i}$: Change in velocity of i^th^ player
$\:{R}_{i}$: Rank of i^th^ player
$\:{X}_{i}$: Position of i^th^ player
$\:mean\left(X\right)$: Mean position of the opposite team
$$\:{r}_{1}$$: Random value $$\:\in\:(0,\:1)$$
$\:Lead\:A$, $\:Lead\:B$: Leaders of the teams A and B
$\:{Lead}_{Op}$: Leader of the opposite team
$\:{X}_{rand}$: Rand of the opposite team
$$\:{r}_{2}\:\&\:{r}_{3}$$: Random values $$\:\in\:(0,\:1)$$
$$\:Max-iter$$: Maximum iteration
$$\:{S}_{i}$$: Success score
Lb: Lower bound
Ub: Upper bound
